# Congenital myasthenic syndrome in Golden Retrievers is associated with a novel *COLQ* mutation

**DOI:** 10.1111/jvim.15667

**Published:** 2019-11-26

**Authors:** Kate L. Tsai, Karen M. Vernau, Kathryn Winger, Danielle M. Zwueste, Beverly K. Sturges, Marguerite Knipe, D. Colette Williams, Kendall J. Anderson, Jacquelyn M. Evans, Ling T. Guo, Leigh Anne Clark, G. Diane Shelton

**Affiliations:** ^1^ Department of Genetics and Biochemistry Clemson University Clemson South Carolina; ^2^ Department of Surgical and Radiological Sciences School of Veterinary Medicine, University of California Davis Davis California; ^3^ William R. Pritchard Veterinary Medical Teaching Hospital, University of California Davis Davis; ^4^ Cancer Genetics and Comparative Genomics Branch, National Human Genome Research Institute, National Institutes of Health Bethesda Maryland; ^5^ Department of Pathology School of Medicine, University of California San Diego La Jolla California

**Keywords:** canine, myasthenia gravis, myopathy, neuromuscular junction

## Abstract

**Background:**

Congenital myasthenic syndromes (CMSs) are a group of inherited disorders of neuromuscular transmission that may be presynaptic, synaptic, or postsynaptic. Causative mutations have been identified in 4 breeds including the Labrador Retriever, Jack Russell Terrier, Heideterrier, and Danish Pointing Dog.

**Hypothesis/Objective:**

Clinical and genetic characterization of a neuromuscular disorder in Golden Retriever (GR) puppies.

**Animals:**

Four GR puppies from California were evaluated for generalized muscle weakness beginning at weaning. Biological specimens were collected from the affected puppies, and familial information was obtained. Blood or buccal swabs were obtained from 63 unaffected GRs.

**Methods:**

Complete physical, neurological, electrodiagnostic, and histological evaluations and biochemical quantification of muscle acetylcholine receptors were performed. Polymerase chain reaction was used to amplify the 17 exons of *COLQ*, and sequences were obtained by Sanger sequencing. Variant frequency was assessed in unrelated GRs and a public database.

**Results:**

Clinical, neurological, and electrodiagnostic evaluations confirmed a disorder of neuromuscular transmission in a GR family. Sequencing of all exons and splice sites of a primary candidate gene, *COLQ*, identified a point mutation that predicts an amino acid substitution (G294R). The primary *COLQ* transcript was absent from affected muscle samples. All affected puppies were homozygous for the mutation, which was not detected outside this GR family or in other breeds.

**Conclusions and Clinical Importance:**

We confirmed the diagnosis of a CMS in GR puppies and identified a novel *COLQ* mutation. The *COLQ* gene encodes the collagenous tail of acetylcholinesterase, the enzyme responsible for termination of skeletal muscle contraction by clearing acetylcholine at the neuromuscular junction. Clinicians and breeders should be aware of this CMS in GR puppies with an early onset of weakness.

**Abbreviations**
AChR: acetylcholine receptor
CHRNE: gene encoding the epsilon subunit of the nicotinic acetylcholine receptor
CMAP: compound muscle action potential
CMS: congenital myasthenic syndrome
COLQ: gene encoding the collagenous tail of acetylcholinesterase
EMG: electromyography
GR: Golden Retriever
MG: myasthenia gravis
PCR: polymerase chain reaction

## 1 INTRODUCTION

Congenital myasthenic syndrome (CMS) refers to a heterogenous group of inherited disorders of neuromuscular transmission that may be presynaptic, synaptic or post‐synaptic.^1^ To date, 32 CMSs have been described in people with autosomal recessive and autosomal dominant modes of inheritance.^2^ Clinical signs of CMS in dogs usually begin at weaning with progressive muscle weakness that is exacerbated by exercise. Unlike in acquired myasthenia gravis (MG) in dogs, megaesophagus is not a common clinical feature.

Although the first clinical report of congenital MG in dogs was published in 1974,^3^ only a few gene mutations causing CMS have been identified. These include a missense mutation in the gene encoding choline acetyltransferase in Danish Pointing dogs,^4^ a nonsynonymous mutation in the gene encoding the collagen‐like tail of the asymmetric acetylcholinesterase (*COLQ*) in Labrador Retrievers,^5^ and a deletion and nonsynonymous mutation in the gene encoding the epsilon subunit of the nicotinic acetylcholine receptor (*CHRNE)* in Jack Russell Terriers^6^ and in Heideterriers,^7^ respectively. Congenital myasthenic syndromes also have been clinically described in Smooth Fox terriers^8^, ^9^ and English Springer Spaniels,^10^ but mutations have not yet been described.

Over a 2‐year period, 4 Golden Retriever (GR) puppies from California were examined for neuromuscular weakness beginning at the time of weaning. Other than exercise intolerance, the puppies were clinically normal. Confirming a diagnosis of a CMS can be difficult, and a presumptive diagnosis can be made from the age of onset, neurological examination, electrodiagnostic testing, and muscle biopsy to rule out other congenital neuromuscular diseases. Where available, genetic testing can confirm the diagnosis. Identification of new CMSs and development of new genetic tests would be useful for the diagnosis of these disorders. Here we describe a novel *COLQ* mutation in GR puppies associated with CMS.

## 2 MATERIALS AND METHODS

### 2.1 Animals

Four related GR puppies, 2 males and 2 females, ranging from 3 to 5 months of age, were evaluated. All puppies were from the same breeder in Southern California. Parentage information and pedigrees could be obtained for only 2 of the affected puppies. Unaffected GRs (n = 63) had no known relationships with this family.

### 2.2 Electrodiagnostic testing

One affected pup was anesthetized, and complete neuromuscular examinations were performed including electromyography (EMG), measurement of motor and sensory nerve conduction velocities, and measurement of the compound muscle action potential (CMAP) after repetitive nerve stimulation at 1, 3, 10, and 50 Hz using a Nicolet Viking Select EMG/evoked potential system (Nicolet, Biomedical Inc, Madison, WI). Insulated stainless steel needle electrodes were used for both nerve stimulation and recording from muscle, and a platinum subdermal electrode (Grass‐Telefactor) was employed as a ground. Motor nerve conduction velocity of the peroneal and ulnar nerves was determined by dividing the distance between proximal and distal stimulation sites by the difference in latency of the corresponding CMAP recorded from the extensor digitorum brevis^11^ and palmar interosseous^12^ muscles, respectively, after supramaximal stimulation (2 Hz stimulus rate, 0.2 millisecond stimulus duration). Sensory nerve conduction studies were performed on the peroneal, ulnar, and radial nerves using previously described techniques.^13^, ^14^ Amplitude (peak‐to‐peak) was measured from CMAPs, and percentages of decrement were calculated for each repetition rate. Muscle (vastus lateralis, triceps brachii, and cranial tibial) and nerve (peroneal nerve) biopsy specimens were collected from the side opposite that used for electrodiagnostic testing.

### 2.3 Histopathology, histochemistry, and fluorescence microscopy

Diagnostic muscle biopsy specimens were collected under general anesthesia after electrodiagnostic testing. Immediately after collection, muscles were snap frozen in isopentane precooled in liquid nitrogen and stored at −80°C until further processing. Light microscopic evaluation of histological and histochemical stains and reactions was performed according to standard protocols^15^ and included hematoxylin and eosin, modified Gomori trichrome, periodic acid Schiff, phosphorylase, esterase, myofibrillar ATPase reactions with preincubation pH of 9.8 and 4.3, reduced nicotinamide adenine dinucleotide‐tetrazolium reductase, succinic dehydrogenase, acid and alkaline phosphatase, and oil red O.

Specimens from the peroneal nerve were immersion fixed in 2.5% glutaraldehyde in 0.1 M phosphate buffer before shipment to an author's laboratory (G.D.S.). Upon receipt, nerves were postfixed in 1% aqueous osmium tetroxide for 3 to 4 hours and then dehydrated in a graded alcohol series and propylene oxide. After infiltration with a 1:1 mixture of propylene oxide and araldite resin for 4 hours, nerves were placed in 100% araldite resin overnight and then embedded in fresh araldite resin. Thick sections (1 μm) were cut and stained with paraphenylediamine before light microscopic evaluation.

For histochemical localization of motor end‐plates, serial cryosections (8 μm) were obtained from the external intercostal muscle of 1 affected GR collected at necropsy. In addition, archived frozen muscle of a previously diagnosed Jack Russell Terrier with CMS caused by end‐plate acetylcholine receptor (AChR) deficiency (neuromuscular disease control)^6^ and archived normal dog intercostal muscle (wild‐type control) were included. Sections from each dog were incubated with the esterase reaction^15^ for identification of presumptive motor end‐plates or Alexa Fluor 594 α‐bungarotoxin (1:1000, Molecular Probes) for localization of AChRs at the motor end‐plate according to published procedures.^6^ Serial sections were evaluated by light microscopy (esterase reaction) or fluorescent microscopy (red fluorescence) followed by localization of staining for comparison.

### 2.4 Sample collection and nucleic acid isolation

Muscle biopsy specimens were collected from an affected GR puppy, and whole blood was obtained from all 4 affected puppies. Blood or buccal swabs were obtained from unaffected GRs. Informed owner consent was obtained before collection of all biologic samples, in accordance with protocols approved by the Clemson University Institutional Review Board (IBC2008‐17). Owner consent was obtained for necropsy and postmortem tissue collection from 1 affected puppy at University of California, Davis.

Genomic DNA was isolated from blood and buccal swabs using Gentra Puregene Blood Kit (Qiagen, Germantown, Maryland) and from muscle using the DNEasy Kit (Qiagen). Ribonucleic acid was extracted from snap frozen muscle tissue from an affected dog using the ToTALLY RNA kit (Ambion, Foster City, California). Canine testis RNA was obtained from Zyagen (San Diego, California). All RNAs were treated with the Turbo DNA‐free kit (Invitrogen, Carlsbad, California), and cDNAs were synthesized using an oligo dT primer and the RevertAid First Strand cDNA Synthesis kit (Thermo Scientific, Pittsburg, Pennsylvania).

### 2.5 Sequencing and genotyping

Polymerase chain reactions (PCRs) to amplify the 17 exons of genomic *COLQ* were performed using previously described primers and protocols.^5^ Primers to amplify the last 7 exons of the *COLQ* transcript were designed with the forward primer (5′ GGGCAGAAAGGTGAAATGGGT) spanning the exon 10 and 11 junction and the reverse primer located in exon 17 (5′ ATGTGAAGTAGCGGCAGGAC). A constitutively expressed gene (*PSMB7*) was used to ensure that cDNA was present. Amplicons were reamplified using band‐stab PCR. Products were purified using an ExoSAP master mix consisting of 0.5 units of Exonuclease I (New England BioLabs, Ipswich, Massachusetts) and 0.25 units of shrimp alkaline phosphatase (SAP, Promega, Madison, Washington). Sequencing products were resolved on an ABI 3730XL DNA Analyzer (Applied Biosystems, Foster City, California). The c.880 G>A variant was genotyped in the remaining 3 affected puppies and 63 unaffected GRs using Sanger sequencing. We further investigated the presence of the variant in a variant call file containing single nucleotide polymorphisms and small insertions and deletions identified in the genomes of 668 domestic dogs, including 20 purebred GR, and 54 wild canids.^16^ In silico programs, PolyPhen‐2^17^ and CPD prediction tool,^18^ were used to assess the impact of the variant.

### 2.6 AChR quantification and antibody‐bound AChR

Acetylcholine receptors were extracted from an external intercostal muscle specimen collected at necropsy from an affected puppy using a modification of a previously described procedure.^19^ The muscle specimens were stored at −70°C before homogenization and extraction of AChR in 2% Triton X‐100. Solubilized AChRs were labeled by incubation with an excess of ^125^I‐α‐bungarotoxin (^125^I‐αbgt) followed by sequential addition of high titer rat‐anti‐AChR antibody and precipitation with goat anti‐rat immunoglobulin G (IgG). The precipitate was pelleted, washed, and quantitated in a gamma counter. The amount of AChR complexed with ^125^I‐αbgt was quantified and expressed in terms of moles of ^125^I‐αbgt precipitated per gram of tissue. The concentration of in situ antibody‐AChR complexes was determined by precipitation with anti‐dog IgG in the presence of normal dog serum. Quantitative serum AChR antibody concentrations were determined as previously described using an immunoprecipitation radioimmunoassay procedure.^19^

## 3 RESULTS

### 3.1 Clinical description

Four related puppies were presented over a 2‐year period to the University of California Davis Veterinary Medical Teaching Hospital Neurology/Neurosurgery Service for clinical evaluation of generalized weakness since approximately 6‐8 weeks of age. The pups occasionally would have a burst of energy but would shortly thereafter sit or lie down. No abnormalities were noted on physical examinations of 3 of the 4 puppies. One puppy was bilaterally cryptorchid with stridor at rest that worsened with excitement or activity. This puppy also had a history of regurgitation. On neurological examination, abnormalities were restricted to an abnormal gait in all puppies. The pups were ambulatory with a choppy gait that became shorter and stiffer with continued walking to the point that the pups would lie down (Video S1). The pups appeared to tire easily and sat down often during examination. Clinical signs were most consistent with generalized neuromuscular disease caused by neuropathy, myopathy, or disorder of neuromuscular transmission. Based on the young age and multiple related animals affected, an inherited neuromuscular disorder was considered most likely. CBC and serum biochemical analysis, including serum creatine kinase activities, were within normal limits for puppies. Thoracic radiographs were normal in 1 puppy. An edrophonium chloride challenge was performed in 2 dogs. In 1 dog, there was no clinical response, and in the other dog the challenge resulted in rapid worsening of clinical signs to the point of collapse, with gradual improvement over a few minutes to baseline.

All 4 puppies were treated with albuterol 0.5 mg PO q8h (0.02‐0.08 mg/kg), with clinical improvement reported by all owners. The 1 puppy with stridor noted on initial examination at 5 months of age presented again at 11 months of age for acute airway obstruction. Laryngeal examination confirmed laryngeal paralysis, and a left laryngeal tieback surgery was performed. Two days after the surgery, the dog developed aspiration pneumonia and died. Of the remaining puppies, 1 puppy was lost to follow‐up at 7 months of age, and 2 puppies were euthanized at 6 months of age. Necropsy was performed on the latter 2 puppies, and no histological abnormalities were found in peripheral nerves, muscle, brain, spinal cord, or other organs.

### 3.2 Electrodiagnostic testing

**Figure 1:**
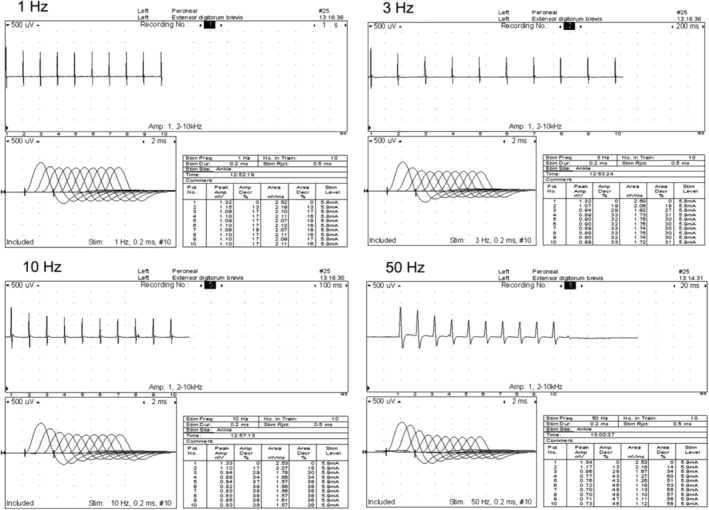
A series of recordings obtained using various repetition rates (1, 3, 10, and 50 Hz) during a repetitive nerve stimulation study in one affected dog. A 1‐minute rest period was utilized between each train of stimuli. All frequencies resulted in decremental responses identical to those associated with myasthenia gravis (a change in amplitude of 10% or greater between the first and subsequent responses). This was the case even with a tetanizing stimulus of 50 Hz when pseudofacilitation (an incremental response in amplitude with little or no change in area) is a typical finding in normal dogs

Abnormalities were restricted to a mild decrease in the amplitude of the M wave, and a decremental response of the CMAP after repetitive nerve stimulation. These findings were consistent with a disorder of neuromuscular transmission (Figure 1). Biopsy specimens from the vastus lateralis, triceps brachii, and cranial tibial muscles were collected under anesthesia for histological evaluation.

### 3.3 Muscle histology, histochemistry, and fluorescence microscopy

**Figure 2:**
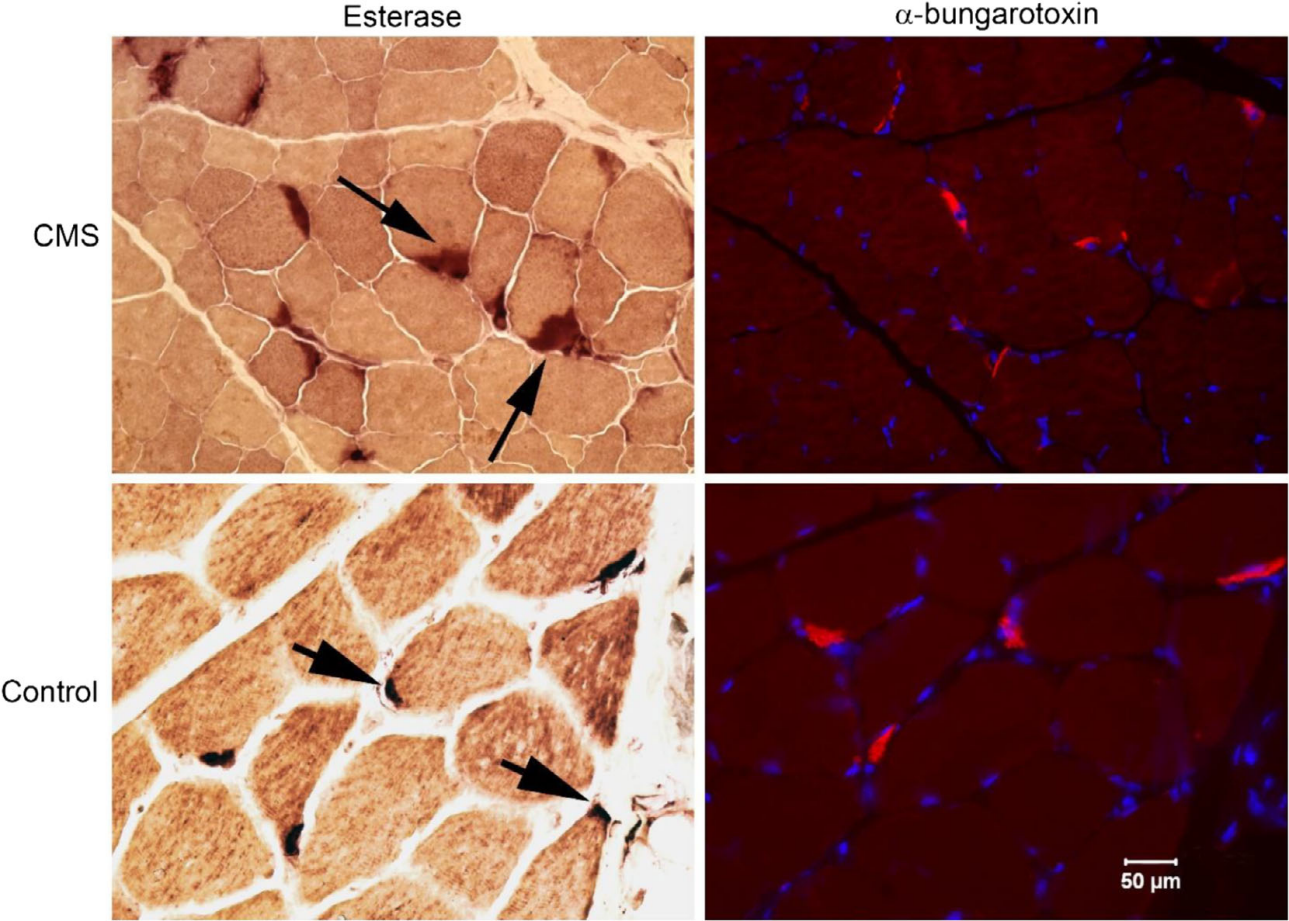
Esterase reaction for localization of motor end‐plates and fluorescent labeled α‐bungarotoxin for localization of acetylcholine receptors (AChRs) in cryosections of muscle from a GR with CMS and archived control muscle. Esterase staining showed enlarged motor end‐plates (arrows with long tail) compared to control muscle (arrows with short tail). AChRs were decreased (red fluorescence) but not absent in the affected puppy compared to control muscle, consistent with the biochemical findings. Dapi staining highlights muscle nuclei. Bar in lower right figure = 50 μm for all images

No specific pathological changes were identified in the muscle or peripheral nerve biopsy specimens that would support an underlying myopathy or neuropathy. The esterase reaction identified several abnormally large motor end‐plates in the affected puppy compared to control end‐plates (Figure 2). Using fluorescent labeled α‐bungarotoxin, staining of motor end‐plates for AChRs was decreased but not absent in the affected puppy compared to control muscle (Figure 2).

### 3.4 AChR quantification

The AChR concentration from 1 affected GR puppy was determined in external intercostal muscle samples collected origin to insertion after euthanasia. The AChR concentration was decreased at 0.11 pmol/g tissue (reference range, 0.2‐0.4 pmol/g tissue). Antibodies were not detected against AChRs in muscle or in serum.

### 3.5 Sequencing and genotyping

**Figure 3:**
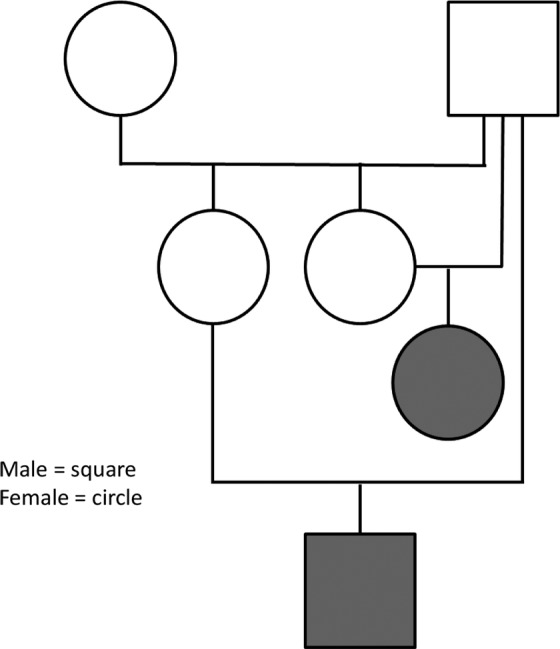
Pedigree for two affected puppies (shaded) shows extensive inbreeding. The affected puppies share a sire that is also the grandsire on the maternal side for both puppies

**Figure 4:**
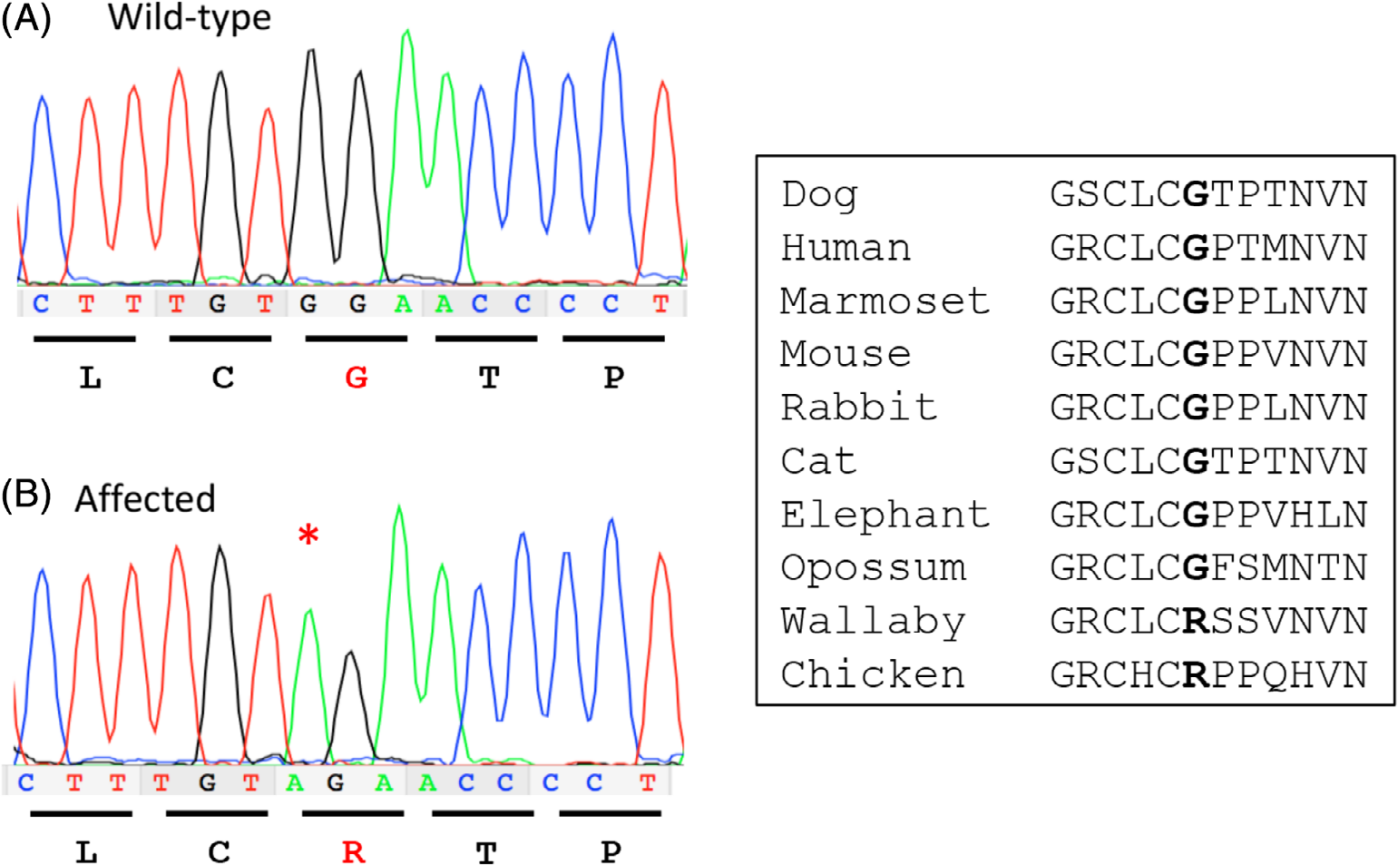
A, Chromatogram from a small section of *COLQ* exon 16 from a control GR (top) and an affected GR (bottom). The G>A mutation predicting a Gly>Arg amino acid change is indicated with a red asterisk (*). B, A partial amino acid sequence of *COLQ* surrounding the predicted substitution is shown from several species. The Glycine (G) that is changed to an Arginine in the affected GR puppies is shown in bold and is conserved across mammals but not chicken or wallaby

Because 1 affected puppy had a decreased (but not absent) concentration of AChRs at the motor end‐plates and did not improve after IV administration of edrophonium chloride, we hypothesized that CMS could be the result of an end‐plate AChE deficiency and prioritized *COLQ* as a candidate causal gene. Evaluation of pedigree information from 2 affected puppies identified extensive inbreeding (Figure 3), suggesting homozygosity for a recessive allele inherited identically by descent. Sequences from 1 affected GR indicated that 16 of 17 *COLQ* exons were concordant with the Boxer reference genome. Exon 13 harbored a G>A transition (chr23:27175559) in position c.880 of *COLQ*. All 4 affected dogs were homozygous for the mutation. The variant was absent from 83 GRs and 702 additional domestic dog and wild canid genomes. The nucleotide change predicts the replacement of a glycine with an arginine (G294R, Figure 4A) in a position conserved across placental mammals (Figure 4B). PolyPhen‐2 predicts the variant to be benign likely because arginine is the wild‐type residue in some organisms (eg, wallaby, chicken). A second in silico prediction program indicates that the arginine may be pathogenic in dogs but compensated by additional COLQ amino acid changes in other organisms (8.1% likelihood).^18^

**Figure 5:**
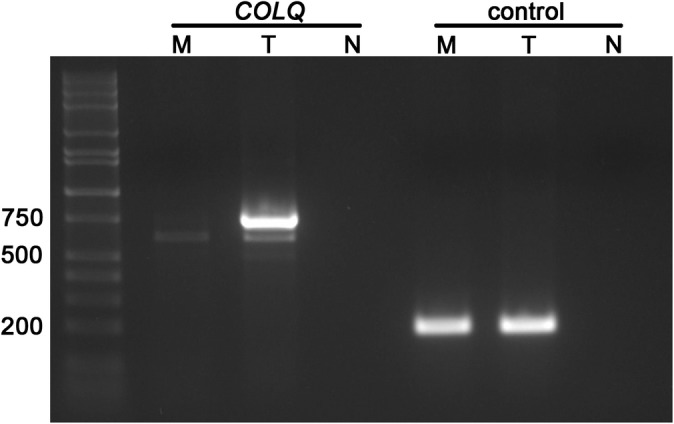
Agarose gel electrophoresis of cDNA amplicons from *COLQ* and a housekeeping control. The muscle of an affected GR (M) lacks the primary 748 bp *COLQ* amplicon present in testis from a control dog (T). N denotes a cDNA negative control. Selected fragment sizes (in bp) for the molecular ladder are marked to the left

The G>A transition changes a GG to AG within exon 13 that could create a new splice acceptor site and lead to aberrant splicing. Amplification and sequencing of partial cDNAs showed 2 isoforms in the testis control: a primary signal containing all exons 11 through 17 (748 bp) and a minor signal that skips exon 13 (608 bp). Complementary DNAs from muscle tissues of an affected dog amplified only the minor signal (Figure 5).

## 4 DISCUSSION

We confirmed a novel CMS in GRs and identified a second mutation in canine *COLQ*. Treatment of CMS can be difficult and requires treatment specific to the gene mutated.^20^ Treatments used for primary AChR deficiency may not be beneficial or may even be detrimental in other forms of CMS.^1^ Because no response was observed to an edrophonium chloride challenge and primary AChR deficiency was not suspected, the puppy was treated with albuterol (0.05 mg/kg PO q8h), a selective β_2_‐adrenergic agonist that has been used in human patients with CMS and end‐plate acetylcholinesterase deficiency.^1^, ^21^, ^22^ The owners reported dramatic improvement in clinical signs in the short term, but eventually the clinical signs progressed despite treatment, resulting in death or euthanasia in the 3 puppies for which the outcome was known.

Clinical differences should be apparent between CMS caused by variants in the *COLQ* and *CHRNE* genes. Although fatigability is present with mutations in both genes, challenge with the short‐acting cholinergic agonist edrophonium chloride or treatment with the longer acting pyridostigmine bromide results in improved muscle strength in dogs with the *CHRNE* mutation^5^ but no response or worsening of muscle weakness after cholinergic agonists in dogs with the *COLQ* variant. In end‐plate AChR deficiency associated with mutations in *CHRNE*, the safety margin of neuromuscular transmission is compromised by a decreased response to acetylcholine because of low AChR content. Cholinergic agonists inhibit AChE and prolong the action of available acetylcholine, thereby improving the safety margin of neuromuscular transmission.^1^ The AChR content is only mildly decreased in *COLQ* variants, and no further benefit can be derived from increasing available acetylcholine. The mechanism by which adrenergic agonists improve neuromuscular transmission is not understood.^1^

Esterase histochemistry similarly localizes motor end‐plates with variants in both genes. Staining for AChRs with fluorescein labeled α‐bgt, however, was not detectable in dogs with the *CHRNE* mutation,^5^ and was decreased but detectable compared to control muscle in the puppies with the *COLQ* mutation. Consistent with this finding, the decremental responses of the CMAP to repetitive nerve stimulation were more marked in the *CHRNE* mutation (decrements of the CMAP of up to 66%)^5^ compared to mild decreases in the dogs with the *COLQ* mutation.

As found in the Labrador Retrievers^4^ and Devon Rex and Sphynx cats^23^ with end‐plate AChE deficiencies, the GR G>A transition causes a missense in the COOH‐terminal domain. COLQ anchors the catalytic subunits of asymmetric AChE to the synaptic basal lamina and stops prolonged muscle contraction by clearing acetylcholine after muscle contraction. The mutation may result in impaired protein‐protein interactions necessary for anchoring COLQ to the basal lamina and may lead to the enlarged and widespread esterase staining found at the motor end‐plates of an affected dog (Figure 2).

The G>A transition in the GR creates a novel AG sequence within exon 13 that we hypothesize could serve as a splice acceptor and cause aberrant splicing. Amplification of cDNAs from affected muscle did not identify abnormal transcripts but rather a complete lack of the primary *COLQ* isoform. A minor isoform that lacks exon 13 was present. This isoform, which creates a frameshift, also was present in the control tissue and has been previously described in humans.^24^

The mechanism behind the absence of COLQ transcripts in the affected dog remains unclear. If the G>A transition does create an alternative splice acceptor, it would cause the loss of 140 nucleotides and a frameshift, likely triggering nonsense‐mediated decay. Alternatively, our approach could have missed an intronic mutation that impacts the cDNA before exon 11 and triggers nonsense‐mediated decay. Regardless of the mechanism, the lack of COLQ transcripts is consistent with the severe CMS phenotype observed in the GRs.

## CONFLICT OF INTEREST DECLARATION

Authors declare no conflict of interest.

## OFF‐LABEL ANTIMICROBIAL DECLARATION

Authors declare no off‐label uses of antimicrobials.

## INSTITUTIONAL ANIMAL CARE AND USE COMMITTEE (IACUC) OR OTHER APPROVAL DECLARATION

Clemson University Institutional Review Board (IDC2008‐17) for DNA processed at Clemson. Owner consent was obtained for clinical necropsy and postmortem tissue collection from UC Davis, therefore no IACUC or other approval was needed for clinical evaluation and diagnostic testing.

## HUMAN ETHICS APPROVAL DECLARATION

Authors declare human ethics approval was not needed for this study.

## Supporting information


**Video S1**
